# Crystal structure of bis(bis{(*E*)-[(6-{(*E*)-[(4-fluorobenzyl)imino]methyl}pyridin-2-yl)methylidene](4-fluorophenyl)amine}nickel(II)) tetra­bromide nona­hydrate

**DOI:** 10.1107/S2056989015021519

**Published:** 2015-11-21

**Authors:** Ismet Basaran, Md Mhahabubur Rhaman, Douglas R. Powell, Md. Alamgir Hossain

**Affiliations:** a Department of Chemistry, Balikesir University, 10145, Balikesir, Turkey; b Department of Chemistry and Biochemistry, Jackson State University, Jackson, MS, 39212, USA; c Department of Chemistry & Biochemistry, University of Oklahoma, 101 Stephenson Parkway, Norman, OK, 73019, USA

**Keywords:** crystal structure, nickel(II) complex, octa­hedral geometry, Schiff base, π–π inter­actions, pyridine derivatives

## Abstract

In the title complex, [Ni(C_21_H_17_F_2_N_3_)_2_]_2_Br_4_·9H_2_O, there are two independent metal complexes per asymmetric unit and two ligands per metal complex. The structural features (bond lengths and angles) of the two complexes are almost identical. In each complex, the nickel(II) ion is coordinated in an octa­hedral environment by six N atoms from two chelating (9*E*)-*N*-({6-[(*E*)-(4-fluoro­benzyl­imino)­meth­yl]pyridin-2-yl}methyl­ene)(4-fluoro­phen­yl)methanammine ligands. The Ni—N bond lengths range from 1.973 (2) to 2.169 (2) Å, while the chelate N—Ni—N angles range from 77.01 (10) to 105.89 (9)°. Additionally, there are four bromide anions and nine solvent water mol­ecules within the asymmetric unit. The water mol­ecules form a hydrogen-bonded network, displaying C—H⋯O, C—H⋯Br, O—H⋯Br, O—H⋯O and O—H⋯F inter­actions into layers parallel to (111). In each unit, the fluoro­phenyl rings of one ligand are stacked with the central ring of the other ligand *via* π–π inter­actions, with the closest centroid-to-plane distances being 3.445 (5), 3.636 (5), 3.397 (5) and 3.396 (5) Å.

## Related literature   

For general background to coordination complexes with Schiff bases, see: Vigato & Tamburini (2004 ▸); Gupta & Sutar (2008 ▸). For applications and bioactivity of metal complexes, see: Skyrianou *et al.* (2010 ▸). For related structures, see: You *et al.* (2014 ▸). For the preparation, see: Işıklan *et al.*, 2011 ▸).
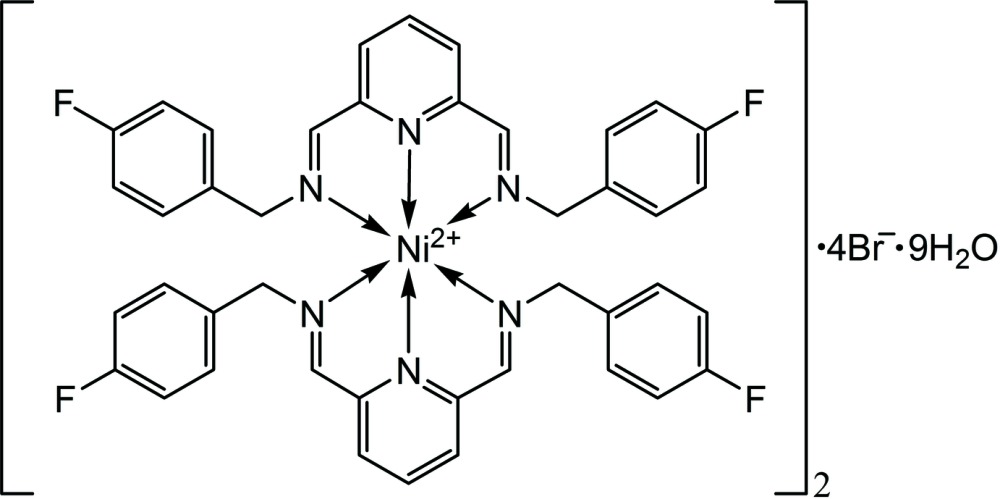


## Experimental   

### Crystal data  

[Ni(C_21_H_17_F_2_N_3_)_2_]_2_Br_4_·9H_2_O
*M* *_r_* = 1996.70
Triclinic, 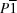

*a* = 12.1000 (16) Å
*b* = 12.5686 (16) Å
*c* = 29.149 (4) Å
α = 87.377 (2)°
β = 86.955 (2)°
γ = 72.339 (2)°
*V* = 4216.0 (10) Å^3^
*Z* = 2
Mo *K*α radiation
μ = 2.42 mm^−1^
*T* = 100 K
0.26 × 0.20 × 0.12 mm

### Data collection  

Bruker APEX CCD diffractometer
Absorption correction: multi-scan (*SADABS*; Krause *et al.*, 2015 ▸) *T*_min_ = 0.571, *T*_max_ = 0.760
94344 measured reflections
19210 independent reflections
15090 reflections with *I* > 2σ(*I*)
*R*_int_ = 0.058

### Refinement  

*R*[*F* ^2^ > 2σ(*F* ^2^)] = 0.042
*wR*(*F* ^2^) = 0.106
*S* = 1.01
19210 reflections
1126 parameters
27 restraints
H atoms treated by a mixture of independent and constrained refinement
Δρ_max_ = 0.84 e Å^−3^
Δρ_min_ = −0.77 e Å^−3^

### 

Data collection: *APEX2* (Bruker, 2007 ▸); cell refinement: *SAINT* (Bruker, 2007 ▸); data reduction: *SAINT*; program(s) used to solve structure: *SHELXS97* (Sheldrick, 2008 ▸); program(s) used to refine structure: *SHELXL2014/7* (Sheldrick, 2015 ▸); molecular graphics: *XP* in *SHELXTL* (Sheldrick, 2008 ▸); software used to prepare material for publication: *SHELXL2014/7*.

## Supplementary Material

Crystal structure: contains datablock(s) I, New_Global_Publ_Block. DOI: 10.1107/S2056989015021519/fk2092sup1.cif

Structure factors: contains datablock(s) I. DOI: 10.1107/S2056989015021519/fk2092Isup2.hkl

**Fig. 1.**
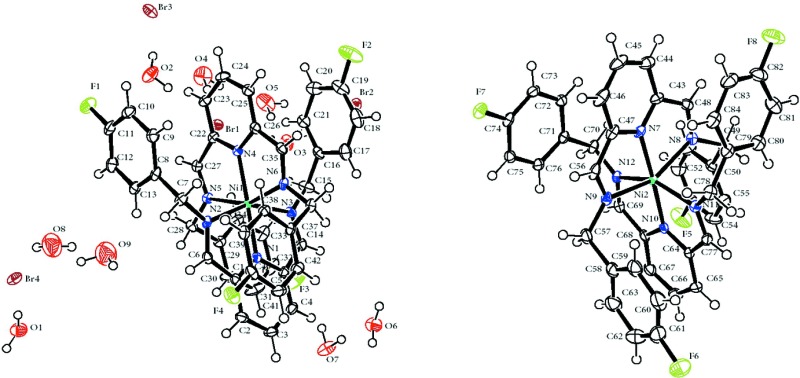
Asymmetric unit of the title compound showing the atom-numbering scheme. Displacement ellipsoids are drawn at the 50% probability level.

**Fig. 2.**
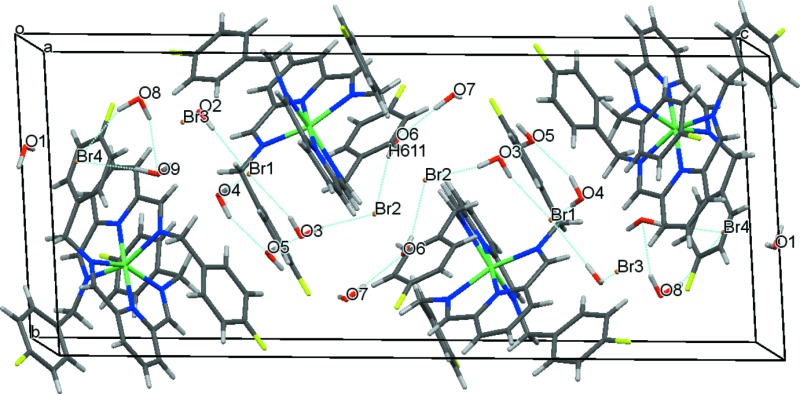
Crystal packing of the title compound viewed along the a axis showing hydrogen-bonding interactions as dashed lines.

. DOI: 10.1107/S2056989015021519/fk2092fig1.tif
Asymmetric unit of the title compound showing the atom-numbering scheme. Displacement ellipsoids are drawn at the 50% probability level.

a . DOI: 10.1107/S2056989015021519/fk2092fig2.tif
Crystal packing of the title compound viewed along the *a* axis showing hydrogen-bonding inter­actions as dashed lines.

**Fig. 3.**
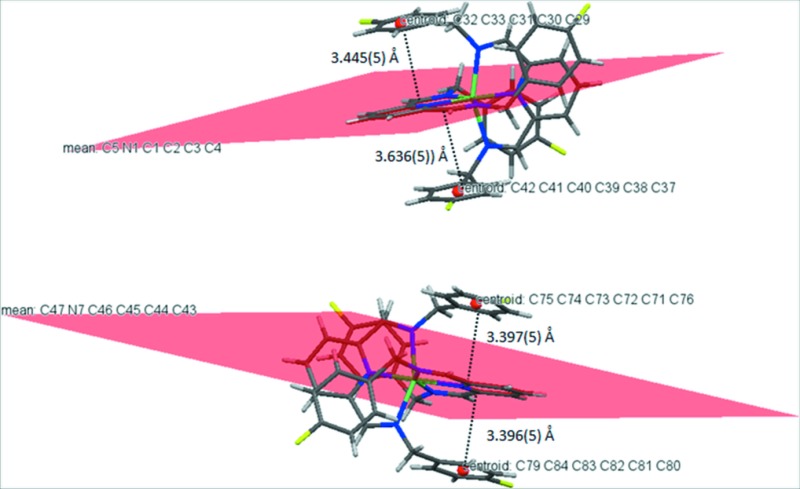
Part of the crystal structure of compound (I) showing the π-π-stacking between fluorophenyl rings and the central pyridine ring.

. DOI: 10.1107/S2056989015021519/fk2092fig3.tif
Part of the crystal structure of compound (I) showing the π-π-stacking between fluoro­phenyl rings and the central pyridine ring.

CCDC reference: 1436891

Additional supporting information:  crystallographic information; 3D view; checkCIF report

## Figures and Tables

**Table 1:** Selected bond lengths (Å)

Ni1—N4	1.984 (2)
Ni1—N1	1.984 (2)
Ni1—N6	2.141 (2)
Ni1—N5	2.151 (2)
Ni1—N2	2.158 (2)
Ni1—N3	2.165 (2)
Ni2—N10	1.973 (2)
Ni2—N7	1.981 (2)
Ni2—N11	2.125 (2)
Ni2—N9	2.158 (2)
Ni2—N12	2.161 (2)
Ni2—N8	2.169 (2)

**Table 2:** Hydrogen-bond geometry (Å, °)

*D*—H⋯*A*	*D*—H	H⋯*A*	*D*⋯*A*	*D*—H⋯*A*
C4—H4⋯O6	0.95	2.60	3.505 (4)	160
C6—H6⋯F7^i^	0.95	2.35	3.238 (3)	155
C18—H18⋯F4^i^	0.95	2.45	3.363 (4)	162
C23—H23⋯O2	0.95	2.56	3.504 (4)	172
C25—H25⋯F2^ii^	0.95	2.43	3.303 (4)	154
C28—H282⋯Br3^iii^	0.99	2.80	3.784 (3)	170
C31—H31⋯O5^iii^	0.95	2.66	3.557 (5)	157
C36—H362⋯Br2^iv^	0.99	2.97	3.933 (3)	164
C39—H39⋯O3^v^	0.95	2.44	3.373 (4)	166
C44—H44⋯F8^vi^	0.95	2.48	3.341 (4)	150
C46—H46⋯Br3^ii^	0.95	2.79	3.708 (3)	164
C52—H52⋯O9^iv^	0.95	2.53	3.292 (6)	138
C60—H60⋯F8^vii^	0.95	2.55	3.329 (4)	139
C62—H62⋯O9^viii^	0.95	2.48	3.365 (6)	156
C65—H65⋯Br4^viii^	0.95	2.91	3.653 (3)	136
C67—H67⋯O2^iv^	0.95	2.58	3.503 (4)	165
C69—H69⋯Br1^iv^	0.95	3.07	3.750 (3)	130
C73—H73⋯F1^ii^	0.95	2.60	3.312 (4)	132
C81—H81⋯F5^ix^	0.95	2.50	3.436 (4)	169
C83—H83⋯O1^x^	0.95	2.52	3.263 (4)	135
O1—H111⋯Br4^xi^	0.89 (2)	2.57 (2)	3.449 (3)	167 (3)
O1—H112⋯Br4	0.88 (2)	2.61 (2)	3.476 (3)	170 (3)
O2—H211⋯Br3	0.89 (2)	2.50 (2)	3.384 (3)	168 (3)
O2—H212⋯Br1	0.87 (2)	2.44 (2)	3.317 (2)	178 (3)
O3—H311⋯Br2	0.90 (2)	2.39 (2)	3.274 (2)	169 (3)
O3—H312⋯Br1	0.89 (2)	2.44 (2)	3.307 (2)	164 (3)
O4—H411⋯O5	0.95 (2)	2.12 (2)	3.033 (5)	161 (4)
O4—H412⋯Br1	0.93 (2)	2.32 (2)	3.250 (3)	178 (4)
O5—H511⋯O3	0.88 (2)	1.93 (2)	2.809 (4)	172 (5)
O5—H512⋯O7^xii^	0.90 (2)	2.11 (3)	2.906 (4)	147 (4)
O6—H611⋯Br2^iv^	0.86 (2)	2.57 (2)	3.407 (2)	165 (3)
O6—H612⋯Br2^iii^	0.87 (2)	2.44 (2)	3.306 (3)	173 (4)
O7—H711⋯O6	0.90 (2)	2.03 (2)	2.925 (4)	171 (4)
O8—H811⋯O9	0.94 (2)	2.45 (3)	3.358 (6)	163 (4)
O8—H812⋯Br4	0.94 (2)	2.71 (3)	3.579 (3)	154 (4)

Symmetry codes: (i) 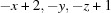; (ii) 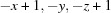; (iii) 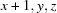; (iv) 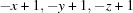; (v) 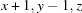; (vi) 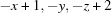; (vii) 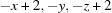; (viii) 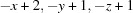; (ix) 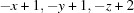; (x) 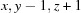; (xi) 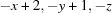; (xii) 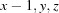.

## supplementary crystallographic information

### S1. Chemical context

Schiff bases are the condensation product of an amine and an active carbonyl compound which are capable of forming coordination complexes with transition metal ions (Vigato *et al.*, 2004; Gupta *et al.*, 2008). In particular, metal complexes derived from Schiff bases with multiple binding sites are effective in many biochemical and anti­microbial applications (Skyrianou *et al.*, 2010).

### S2. Structural commentary

The reported crystal structure of Ni(II) complex with 2,6-di­formyl­pyridine based Schiff base, contains two metal complexes per asymmetric unit. Each metal complex is formed with two ligands, providing almost identical structure to each other (Fig. 1). In each unit, the nickel(II) ion is o­cta­hedrally coordinated with two chelating ligands. The Ni—N bond lengths vary from 1.973 (2) to 2.169 (2) Å, while the N—Ni—N angles are in the range from 77.01 (10) to 105.89 (9) ° (Table 1). Similar coordination pattern of of nickel(II) is reported in the literature (You *et al.* 2014). External bromides and water molecules are liked with the ligands via hydrogen bonding inter­actions with C—H···O, C—H···Br, O—H···Br, O—H···O and O—H···F bonds given in Fig. 2 and Table 2. Weak C—H···F inter­actions are also observed (3.238 (3) to 3.436 (4) Å). In each unit, two fluoro­phenyl rings of one ligand are stacked with the central pyridine ring of the other ligand *via*π- π inter­actions. In one unit, the distances from the plane of central ring (N1 C1 C2 C3 C4 C5) to the centroids of fluoro­phenyl rings (C29 C30 C31 C32 C33 C34 and C37 C38 C39 C40 C41 C42) are 3.445 (5) and 3.636 (5) Å; while in the other unit, the corresponding distances from the plane of central ring (N7 C43 C44 C45 C46 C47) to the centroids of fluoro­phenyl rings (C71 C72 C73 C74 C75 C76 and C79 C80 C81 C82 C83 C84) are 3.397 (5)and 3.396 (5) Å (Fig. 3).

### S3. Supra­molecular features

Inter­molecular inter­actions form various C—H···O, C—H···Br, O—H···Br, O—H···O and O—H···F bonds (Table 1 and Figure 2). In each unit, there are also π- π inter­actions between the fluoro­phenyl rings of one ligand and the central ring of the other ligand.

### S4. Synthesis and crystallization

2,6-di­formyl­pyridine (0.500 g, 3.70 mmol) and 4-fluoro­benzyl­amine (845.8 µL, 7.40 mmol) were separately dissolved in 10 mL methanol. The two solutions were slowly mixed in 20 mL methanol with constant stirring over 10 minutes at room temperature following the similar method as described earlier (Işıklan *et al.*, 2011). The mixture was further stirred overnight and left at room temperature for 24 hours. White crystalline powder thus formed was filtered and dried at room temperature. Yield: 95 %. Melting point is 89°C.

The nickel complex was obtained by mixing of the ligand (0.349 g, dissolved in 5 mL of methanol) and nickel(II) bromide hydrate (0.1092 g, dissolved in 5 mL of water) in water-methanol (50 mL) over 10 minutes under constant stirring at room temperature. After reducing the solvent to about 10 mL, di­ethyl ether was added dropwise. The precipitate thus obtained was filtered and washed with di­ethyl ether, providing a brownish product (0.3902 g, 92.7 % yield). Single crystal suitable for X-ray analysis was grown from the slow evaporation of the complex (107 mg) dissolved in water-methanol (10 mL, v/v, 1:1) after three weeks.

### S5. Refinement details

The H atoms bonded to carbons were placed in calculated positions and treated as riding, C—H = 0.95 to 0.99 Å, with U_iso_(H) = 1.2U_eq_(C). H atoms bonded to O were restrained to have similar O—H lengths and H—O—H angles.

### Crystal data

**Table:**

[Ni(C_21_H_17_F_2_N_3_)_2_]_2_Br_4_·9H_2_O	*Z* = 2
*M**_r_* = 1996.70	*F*(000) = 2028
Triclinic, *P*1	*D*_x_ = 1.573 Mg m^−^^3^
*a* = 12.1000 (16) Å	Mo *K*α radiation, λ = 0.71073 Å
*b* = 12.5686 (16) Å	Cell parameters from 9955 reflections
*c* = 29.149 (4) Å	θ = 2.2–26.8°
α = 87.377 (2)°	µ = 2.42 mm^−^^1^
β = 86.955 (2)°	*T* = 100 K
γ = 72.339 (2)°	Block, orange
*V* = 4216.0 (10) Å^3^	0.26 × 0.20 × 0.12 mm

### Data collection

**Table:**

Bruker APEX CCD diffractometer	15090 reflections with *I* > 2σ(*I*)
φ and ω scans	*R*_int_ = 0.058
Absorption correction: multi-scan (*SADABS*; Krause *et al.*, 2015)	θ_max_ = 27.4°, θ_min_ = 1.4°
*T*_min_ = 0.571, *T*_max_ = 0.760	*h* = −15→15
94344 measured reflections	*k* = −16→16
19210 independent reflections	*l* = −37→37

### Refinement

**Table:**

Refinement on *F*^2^	Primary atom site location: structure-invariant direct methods
Least-squares matrix: full	Secondary atom site location: difference Fourier map
*R*[*F*^2^ > 2σ(*F*^2^)] = 0.042	Hydrogen site location: difference Fourier map
*wR*(*F*^2^) = 0.106	H atoms treated by a mixture of independent and constrained refinement
*S* = 1.01	*w* = 1/[σ^2^(*F*_o_^2^) + (0.050*P*)^2^ + 5.*P*] where *P* = (*F*_o_^2^ + 2*F*_c_^2^)/3
19210 reflections	(Δ/σ)_max_ = 0.001
1126 parameters	Δρ_max_ = 0.84 e Å^−^^3^
27 restraints	Δρ_min_ = −0.77 e Å^−^^3^

### Special details

**Table:**

Geometry. All e.s.d.'s (except the e.s.d. in the dihedral angle between two l.s. planes) are estimated using the full covariance matrix. The cell e.s.d.'s are taken into account individually in the estimation of e.s.d.'s in distances, angles and torsion angles; correlations between e.s.d.'s in cell parameters are only used when they are defined by crystal symmetry. An approximate (isotropic) treatment of cell e.s.d.'s is used for estimating e.s.d.'s involving l.s. planes.

### Fractional atomic coordinates and isotropic or equivalent isotropic displacement parameters (Å^2^)

**Table:**

	*x*	*y*	*z*	*U*_iso_*/*U*_eq_	
Ni1	0.76325 (3)	0.25409 (3)	0.36931 (2)	0.01149 (8)	
F1	0.58023 (17)	0.02363 (18)	0.19175 (7)	0.0338 (5)	
F2	0.3691 (2)	0.14418 (19)	0.51716 (9)	0.0509 (7)	
F3	0.5998 (2)	0.81422 (17)	0.36303 (9)	0.0558 (7)	
F4	1.32720 (16)	−0.10432 (16)	0.40078 (6)	0.0279 (4)	
N1	0.8897 (2)	0.32331 (19)	0.37240 (8)	0.0138 (5)	
N2	0.9073 (2)	0.13752 (19)	0.33378 (8)	0.0137 (5)	
N3	0.6810 (2)	0.4043 (2)	0.40683 (8)	0.0160 (5)	
N4	0.6434 (2)	0.17671 (18)	0.36630 (8)	0.0123 (5)	
N5	0.6780 (2)	0.32319 (19)	0.30703 (8)	0.0138 (5)	
N6	0.7917 (2)	0.14722 (19)	0.42964 (8)	0.0137 (5)	
C1	0.9936 (2)	0.2743 (2)	0.35249 (9)	0.0141 (6)	
C2	1.0856 (3)	0.3181 (3)	0.35519 (10)	0.0204 (6)	
H2	1.1588	0.2832	0.3405	0.025*	
C3	1.0676 (3)	0.4144 (3)	0.37988 (11)	0.0240 (7)	
H3	1.1291	0.4459	0.3825	0.029*	
C4	0.9591 (3)	0.4647 (3)	0.40086 (10)	0.0212 (7)	
H4	0.9457	0.5303	0.4180	0.025*	
C5	0.8714 (3)	0.4170 (2)	0.39614 (9)	0.0161 (6)	
C6	0.9988 (2)	0.1689 (2)	0.33110 (9)	0.0156 (6)	
H6	1.0679	0.1255	0.3158	0.019*	
C7	0.9170 (3)	0.0262 (2)	0.31722 (10)	0.0178 (6)	
H71	0.9099	−0.0234	0.3438	0.021*	
H72	0.9949	−0.0054	0.3023	0.021*	
C8	0.8260 (3)	0.0279 (2)	0.28343 (10)	0.0173 (6)	
C9	0.7620 (3)	−0.0470 (2)	0.28993 (10)	0.0198 (6)	
H9	0.7743	−0.0964	0.3161	0.024*	
C10	0.6802 (3)	−0.0508 (3)	0.25858 (11)	0.0230 (7)	
H10	0.6382	−0.1036	0.2624	0.028*	
C11	0.6625 (3)	0.0246 (3)	0.22210 (11)	0.0234 (7)	
C12	0.7240 (3)	0.1001 (3)	0.21409 (11)	0.0235 (7)	
H12	0.7102	0.1502	0.1881	0.028*	
C13	0.8074 (3)	0.1005 (3)	0.24532 (10)	0.0194 (6)	
H13	0.8518	0.1509	0.2405	0.023*	
C14	0.7500 (3)	0.4593 (2)	0.41423 (10)	0.0171 (6)	
H14	0.7245	0.5261	0.4310	0.021*	
C15	0.5586 (3)	0.4513 (3)	0.42221 (12)	0.0236 (7)	
H151	0.5114	0.4798	0.3950	0.028*	
H152	0.5512	0.5154	0.4419	0.028*	
C16	0.5110 (3)	0.3684 (2)	0.44844 (10)	0.0178 (6)	
C17	0.5573 (3)	0.3180 (3)	0.48937 (11)	0.0273 (7)	
H17	0.6217	0.3343	0.5013	0.033*	
C18	0.5076 (3)	0.2419 (3)	0.51324 (11)	0.0317 (8)	
H18	0.5376	0.2065	0.5414	0.038*	
C19	0.4154 (3)	0.2208 (3)	0.49476 (13)	0.0321 (8)	
C20	0.3667 (3)	0.2698 (3)	0.45514 (12)	0.0278 (7)	
H20	0.3010	0.2544	0.4439	0.033*	
C21	0.4167 (3)	0.3431 (2)	0.43185 (11)	0.0212 (7)	
H21	0.3856	0.3771	0.4037	0.025*	
C22	0.5732 (2)	0.1985 (2)	0.33093 (10)	0.0141 (6)	
C23	0.4899 (3)	0.1437 (2)	0.32709 (11)	0.0183 (6)	
H23	0.4397	0.1599	0.3020	0.022*	
C24	0.4827 (3)	0.0647 (2)	0.36099 (11)	0.0202 (6)	
H24	0.4266	0.0262	0.3594	0.024*	
C25	0.5571 (3)	0.0417 (2)	0.39727 (10)	0.0177 (6)	
H25	0.5531	−0.0130	0.4204	0.021*	
C26	0.6372 (2)	0.1000 (2)	0.39906 (10)	0.0150 (6)	
C27	0.5976 (2)	0.2817 (2)	0.29794 (10)	0.0150 (6)	
H27	0.5551	0.3038	0.2708	0.018*	
C28	0.7039 (3)	0.4057 (2)	0.27429 (10)	0.0181 (6)	
H281	0.6571	0.4132	0.2468	0.022*	
H282	0.7870	0.3799	0.2643	0.022*	
C29	0.6768 (3)	0.5175 (2)	0.29632 (10)	0.0167 (6)	
C30	0.7634 (3)	0.5661 (3)	0.30277 (12)	0.0264 (7)	
H30	0.8406	0.5305	0.2918	0.032*	
C31	0.7377 (3)	0.6665 (3)	0.32519 (14)	0.0367 (9)	
H31	0.7965	0.7004	0.3296	0.044*	
C32	0.6252 (4)	0.7157 (3)	0.34075 (13)	0.0342 (9)	
C33	0.5371 (3)	0.6716 (3)	0.33478 (11)	0.0253 (7)	
H33	0.4601	0.7085	0.3455	0.030*	
C34	0.5638 (3)	0.5711 (2)	0.31246 (10)	0.0183 (6)	
H34	0.5042	0.5383	0.3081	0.022*	
C35	0.7228 (3)	0.0878 (2)	0.43438 (10)	0.0165 (6)	
H35	0.7268	0.0373	0.4599	0.020*	
C36	0.8771 (2)	0.1368 (2)	0.46492 (10)	0.0164 (6)	
H361	0.8563	0.0967	0.4925	0.020*	
H362	0.8755	0.2122	0.4741	0.020*	
C37	0.9982 (2)	0.0740 (2)	0.44699 (9)	0.0138 (6)	
C38	1.0194 (3)	−0.0315 (2)	0.42838 (9)	0.0152 (6)	
H38	0.9572	−0.0624	0.4267	0.018*	
C39	1.1298 (3)	−0.0916 (2)	0.41240 (9)	0.0168 (6)	
H39	1.1438	−0.1625	0.3993	0.020*	
C40	1.2184 (3)	−0.0457 (3)	0.41611 (10)	0.0192 (6)	
C41	1.2020 (3)	0.0561 (3)	0.43528 (11)	0.0228 (7)	
H41	1.2656	0.0846	0.4380	0.027*	
C42	1.0908 (3)	0.1167 (3)	0.45058 (10)	0.0195 (6)	
H42	1.0780	0.1877	0.4635	0.023*	
Ni2	0.77765 (3)	0.25297 (3)	0.87380 (2)	0.01204 (8)	
F5	0.4807 (2)	0.79732 (17)	0.89071 (8)	0.0506 (7)	
F6	1.3604 (2)	0.2490 (3)	0.88290 (9)	0.0621 (8)	
F7	0.73668 (15)	−0.01445 (15)	0.68867 (6)	0.0238 (4)	
F8	0.6187 (2)	−0.0641 (2)	1.03512 (8)	0.0489 (6)	
N7	0.7323 (2)	0.1156 (2)	0.86789 (8)	0.0152 (5)	
N8	0.6370 (2)	0.2721 (2)	0.92506 (8)	0.0157 (5)	
N9	0.8962 (2)	0.1667 (2)	0.82011 (8)	0.0179 (5)	
N10	0.8291 (2)	0.3870 (2)	0.87726 (8)	0.0145 (5)	
N11	0.9127 (2)	0.2000 (2)	0.92119 (8)	0.0153 (5)	
N12	0.6656 (2)	0.3705 (2)	0.82711 (8)	0.0148 (5)	
C43	0.6403 (3)	0.1026 (2)	0.89251 (10)	0.0169 (6)	
C44	0.6034 (3)	0.0096 (3)	0.88746 (11)	0.0242 (7)	
H44	0.5364	0.0018	0.9040	0.029*	
C45	0.6672 (3)	−0.0719 (3)	0.85746 (12)	0.0296 (8)	
H45	0.6438	−0.1365	0.8536	0.036*	
C46	0.7639 (3)	−0.0596 (3)	0.83341 (12)	0.0278 (7)	
H46	0.8094	−0.1161	0.8137	0.033*	
C47	0.7929 (3)	0.0383 (2)	0.83883 (10)	0.0189 (6)	
C48	0.5905 (3)	0.1932 (2)	0.92464 (10)	0.0177 (6)	
H48	0.5258	0.1932	0.9445	0.021*	
C49	0.5970 (3)	0.3555 (2)	0.96102 (10)	0.0216 (7)	
H491	0.6590	0.3444	0.9832	0.026*	
H492	0.5282	0.3439	0.9779	0.026*	
C50	0.5660 (3)	0.4734 (2)	0.94172 (10)	0.0166 (6)	
C51	0.4689 (3)	0.5170 (3)	0.91533 (11)	0.0243 (7)	
H51	0.4218	0.4715	0.9092	0.029*	
C52	0.4401 (3)	0.6260 (3)	0.89788 (12)	0.0319 (8)	
H52	0.3744	0.6558	0.8796	0.038*	
C53	0.5096 (3)	0.6896 (3)	0.90792 (12)	0.0317 (8)	
C54	0.6044 (3)	0.6514 (3)	0.93415 (13)	0.0308 (8)	
H54	0.6498	0.6982	0.9407	0.037*	
C55	0.6325 (3)	0.5421 (3)	0.95092 (11)	0.0240 (7)	
H55	0.6987	0.5135	0.9691	0.029*	
C56	0.8857 (3)	0.0708 (3)	0.81292 (10)	0.0208 (7)	
H56	0.9362	0.0222	0.7916	0.025*	
C57	0.9801 (3)	0.2055 (3)	0.79100 (10)	0.0240 (7)	
H571	0.9394	0.2783	0.7762	0.029*	
H572	1.0122	0.1515	0.7664	0.029*	
C58	1.0792 (3)	0.2184 (3)	0.81762 (11)	0.0253 (7)	
C59	1.1418 (3)	0.1330 (3)	0.84669 (11)	0.0301 (8)	
H59	1.1198	0.0667	0.8511	0.036*	
C60	1.2355 (3)	0.1433 (4)	0.86923 (12)	0.0381 (9)	
H60	1.2777	0.0854	0.8894	0.046*	
C61	1.2655 (3)	0.2390 (4)	0.86174 (13)	0.0415 (10)	
C62	1.2077 (3)	0.3257 (4)	0.83371 (14)	0.0426 (10)	
H62	1.2314	0.3910	0.8294	0.051*	
C63	1.1116 (3)	0.3148 (3)	0.81146 (12)	0.0342 (8)	
H63	1.0686	0.3740	0.7921	0.041*	
C64	0.9163 (3)	0.3837 (3)	0.90442 (9)	0.0166 (6)	
C65	0.9589 (3)	0.4741 (3)	0.90627 (10)	0.0224 (7)	
H65	1.0196	0.4721	0.9259	0.027*	
C66	0.9101 (3)	0.5674 (3)	0.87855 (11)	0.0266 (7)	
H66	0.9381	0.6303	0.8791	0.032*	
C67	0.8210 (3)	0.5703 (3)	0.85002 (10)	0.0234 (7)	
H67	0.7880	0.6339	0.8308	0.028*	
C68	0.7815 (3)	0.4771 (2)	0.85045 (9)	0.0168 (6)	
C69	0.6881 (3)	0.4634 (2)	0.82323 (10)	0.0172 (6)	
H69	0.6457	0.5220	0.8034	0.021*	
C70	0.5697 (3)	0.3566 (2)	0.80145 (10)	0.0179 (6)	
H701	0.5356	0.4249	0.7824	0.022*	
H702	0.5083	0.3456	0.8233	0.022*	
C71	0.6133 (3)	0.2571 (2)	0.77086 (10)	0.0162 (6)	
C72	0.5662 (3)	0.1685 (3)	0.77556 (10)	0.0189 (6)	
H721	0.5052	0.1712	0.7978	0.023*	
C73	0.6080 (3)	0.0763 (3)	0.74791 (10)	0.0194 (6)	
H73	0.5772	0.0152	0.7512	0.023*	
C74	0.6948 (3)	0.0763 (2)	0.71574 (10)	0.0173 (6)	
C75	0.7429 (3)	0.1627 (3)	0.70934 (10)	0.0177 (6)	
H75	0.8029	0.1599	0.6866	0.021*	
C76	0.7001 (3)	0.2542 (3)	0.73748 (10)	0.0171 (6)	
H76	0.7308	0.3153	0.7337	0.021*	
C77	0.9603 (3)	0.2750 (3)	0.92889 (10)	0.0174 (6)	
H77	1.0215	0.2612	0.9496	0.021*	
C78	0.9627 (3)	0.0873 (2)	0.94129 (10)	0.0196 (6)	
H781	1.0236	0.0882	0.9625	0.024*	
H782	0.9996	0.0353	0.9165	0.024*	
C79	0.8714 (3)	0.0458 (2)	0.96719 (10)	0.0181 (6)	
C80	0.8033 (3)	0.1073 (3)	1.00239 (11)	0.0230 (7)	
H80	0.8154	0.1750	1.0107	0.028*	
C81	0.7179 (3)	0.0710 (3)	1.02553 (12)	0.0288 (8)	
H81	0.6698	0.1135	1.0492	0.035*	
C82	0.7048 (3)	−0.0294 (3)	1.01308 (12)	0.0316 (8)	
C83	0.7727 (3)	−0.0940 (3)	0.98003 (12)	0.0315 (8)	
H83	0.7628	−0.1635	0.9731	0.038*	
C84	0.8569 (3)	−0.0558 (3)	0.95670 (11)	0.0251 (7)	
H84	0.9050	−0.0994	0.9333	0.030*	
Br1	0.32248 (3)	0.43862 (2)	0.30040 (2)	0.02087 (7)	
Br2	0.16973 (3)	0.57593 (3)	0.47672 (2)	0.02491 (8)	
Br3	0.01026 (3)	0.28738 (3)	0.22480 (2)	0.02845 (8)	
Br4	0.87440 (3)	0.36772 (3)	0.04185 (2)	0.02785 (8)	
O1	0.8522 (2)	0.6364 (2)	−0.00019 (9)	0.0345 (6)	
H111	0.923 (2)	0.625 (3)	−0.0133 (13)	0.041*	
H112	0.848 (3)	0.571 (2)	0.0100 (13)	0.041*	
O2	0.2986 (2)	0.2342 (2)	0.23730 (9)	0.0330 (6)	
H211	0.2223 (17)	0.244 (3)	0.2383 (13)	0.040*	
H212	0.303 (3)	0.289 (3)	0.2538 (12)	0.040*	
O3	0.2289 (2)	0.6485 (2)	0.37117 (8)	0.0335 (6)	
H311	0.220 (3)	0.619 (3)	0.3994 (7)	0.040*	
H312	0.265 (3)	0.586 (2)	0.3565 (11)	0.040*	
O4	0.0575 (3)	0.5247 (3)	0.26652 (10)	0.0474 (7)	
H411	0.038 (4)	0.595 (2)	0.2806 (14)	0.057*	
H412	0.133 (2)	0.499 (3)	0.2770 (15)	0.057*	
O5	0.0106 (3)	0.7161 (3)	0.33174 (11)	0.0504 (8)	
H511	0.082 (2)	0.698 (4)	0.3419 (15)	0.060*	
H512	−0.026 (3)	0.769 (3)	0.3519 (13)	0.060*	
O6	0.8831 (2)	0.6656 (2)	0.48592 (9)	0.0317 (6)	
H611	0.860 (3)	0.613 (3)	0.4990 (13)	0.038*	
H612	0.9584 (16)	0.644 (3)	0.4858 (13)	0.038*	
O7	0.8465 (2)	0.8179 (2)	0.40539 (10)	0.0372 (6)	
H711	0.850 (3)	0.773 (3)	0.4306 (10)	0.045*	
H712	0.778 (2)	0.814 (3)	0.3994 (13)	0.045*	
O8	0.9354 (3)	0.1547 (3)	0.13081 (12)	0.0658 (10)	
H811	0.893 (4)	0.221 (3)	0.1447 (15)	0.079*	
H812	0.908 (4)	0.194 (4)	0.1034 (10)	0.079*	
O9	0.7736 (3)	0.4144 (4)	0.15719 (13)	0.0771 (11)	
H911	0.843 (3)	0.379 (5)	0.1739 (15)	0.092*	
H912	0.809 (4)	0.396 (5)	0.1269 (9)	0.092*	

### Atomic displacement parameters (Å^2^)

**Table:**

	*U*^11^	*U*^22^	*U*^33^	*U*^12^	*U*^13^	*U*^23^
Ni1	0.01170 (18)	0.00862 (17)	0.01394 (17)	−0.00256 (14)	−0.00089 (13)	−0.00129 (13)
F1	0.0307 (11)	0.0424 (13)	0.0317 (11)	−0.0136 (10)	−0.0155 (9)	−0.0017 (9)
F2	0.0433 (14)	0.0392 (13)	0.0659 (16)	−0.0118 (11)	0.0065 (12)	0.0281 (12)
F3	0.0698 (17)	0.0147 (11)	0.0826 (18)	−0.0059 (11)	−0.0303 (14)	−0.0155 (11)
F4	0.0182 (9)	0.0332 (11)	0.0268 (10)	−0.0003 (8)	0.0022 (8)	0.0007 (8)
N1	0.0159 (12)	0.0123 (12)	0.0131 (11)	−0.0044 (10)	−0.0006 (9)	0.0007 (9)
N2	0.0167 (12)	0.0106 (12)	0.0119 (11)	−0.0005 (10)	−0.0024 (9)	−0.0021 (9)
N3	0.0165 (12)	0.0129 (12)	0.0176 (12)	−0.0037 (10)	0.0039 (10)	−0.0005 (10)
N4	0.0123 (12)	0.0072 (11)	0.0153 (11)	0.0003 (9)	−0.0001 (9)	−0.0022 (9)
N5	0.0134 (12)	0.0082 (11)	0.0174 (12)	0.0002 (9)	−0.0007 (9)	0.0001 (9)
N6	0.0155 (12)	0.0103 (11)	0.0127 (11)	0.0002 (10)	−0.0017 (9)	−0.0022 (9)
C1	0.0150 (14)	0.0158 (14)	0.0113 (13)	−0.0044 (12)	−0.0014 (11)	0.0012 (11)
C2	0.0153 (15)	0.0273 (17)	0.0203 (15)	−0.0093 (13)	−0.0007 (12)	0.0031 (13)
C3	0.0258 (17)	0.0297 (18)	0.0240 (16)	−0.0198 (15)	−0.0032 (13)	0.0020 (13)
C4	0.0311 (18)	0.0187 (16)	0.0185 (15)	−0.0141 (14)	−0.0031 (13)	−0.0020 (12)
C5	0.0224 (16)	0.0141 (14)	0.0141 (13)	−0.0087 (12)	−0.0028 (11)	0.0011 (11)
C6	0.0127 (14)	0.0183 (15)	0.0131 (13)	−0.0008 (12)	0.0001 (11)	−0.0009 (11)
C7	0.0170 (15)	0.0112 (14)	0.0235 (15)	−0.0006 (12)	−0.0035 (12)	−0.0045 (12)
C8	0.0181 (15)	0.0122 (14)	0.0187 (14)	0.0009 (12)	−0.0018 (12)	−0.0073 (11)
C9	0.0206 (16)	0.0150 (15)	0.0216 (15)	−0.0020 (12)	0.0005 (12)	−0.0033 (12)
C10	0.0230 (17)	0.0188 (16)	0.0283 (17)	−0.0073 (13)	−0.0011 (13)	−0.0060 (13)
C11	0.0217 (17)	0.0265 (17)	0.0222 (16)	−0.0056 (14)	−0.0059 (13)	−0.0088 (13)
C12	0.0284 (18)	0.0209 (16)	0.0187 (15)	−0.0029 (14)	−0.0037 (13)	−0.0008 (12)
C13	0.0218 (16)	0.0161 (15)	0.0199 (15)	−0.0044 (13)	−0.0007 (12)	−0.0055 (12)
C14	0.0248 (16)	0.0099 (14)	0.0159 (14)	−0.0045 (12)	0.0031 (12)	−0.0022 (11)
C15	0.0173 (16)	0.0132 (15)	0.0367 (18)	−0.0009 (12)	0.0081 (13)	0.0005 (13)
C16	0.0192 (15)	0.0117 (14)	0.0185 (14)	−0.0001 (12)	0.0078 (12)	−0.0015 (11)
C17	0.0246 (17)	0.0287 (18)	0.0219 (16)	0.0018 (14)	0.0018 (13)	−0.0033 (14)
C18	0.036 (2)	0.0286 (19)	0.0198 (16)	0.0048 (16)	−0.0014 (14)	0.0073 (14)
C19	0.0271 (19)	0.0267 (19)	0.038 (2)	−0.0040 (15)	0.0097 (15)	0.0078 (15)
C20	0.0218 (17)	0.0196 (17)	0.040 (2)	−0.0043 (14)	0.0019 (15)	−0.0006 (14)
C21	0.0226 (16)	0.0136 (15)	0.0244 (16)	−0.0015 (13)	0.0033 (13)	−0.0009 (12)
C22	0.0127 (14)	0.0096 (13)	0.0176 (14)	0.0004 (11)	−0.0014 (11)	−0.0023 (11)
C23	0.0148 (15)	0.0140 (15)	0.0253 (16)	−0.0021 (12)	−0.0043 (12)	−0.0048 (12)
C24	0.0164 (15)	0.0137 (15)	0.0316 (17)	−0.0059 (12)	0.0002 (13)	−0.0033 (12)
C25	0.0186 (15)	0.0125 (14)	0.0211 (15)	−0.0038 (12)	0.0025 (12)	−0.0008 (11)
C26	0.0130 (14)	0.0119 (14)	0.0174 (14)	0.0001 (11)	0.0036 (11)	−0.0032 (11)
C27	0.0129 (14)	0.0124 (14)	0.0164 (14)	0.0016 (11)	−0.0013 (11)	−0.0016 (11)
C28	0.0209 (16)	0.0157 (15)	0.0167 (14)	−0.0048 (12)	0.0003 (12)	0.0039 (11)
C29	0.0210 (15)	0.0105 (14)	0.0188 (14)	−0.0051 (12)	−0.0048 (12)	0.0054 (11)
C30	0.0209 (17)	0.0217 (17)	0.0383 (19)	−0.0092 (14)	−0.0071 (14)	0.0092 (14)
C31	0.042 (2)	0.0210 (18)	0.056 (2)	−0.0219 (17)	−0.0220 (19)	0.0104 (17)
C32	0.048 (2)	0.0114 (16)	0.042 (2)	−0.0040 (16)	−0.0206 (18)	−0.0002 (14)
C33	0.0315 (19)	0.0120 (15)	0.0283 (17)	0.0008 (13)	−0.0112 (14)	0.0008 (13)
C34	0.0208 (16)	0.0129 (14)	0.0221 (15)	−0.0057 (12)	−0.0081 (12)	0.0045 (12)
C35	0.0186 (15)	0.0130 (14)	0.0163 (14)	−0.0031 (12)	0.0038 (11)	−0.0003 (11)
C36	0.0176 (15)	0.0143 (14)	0.0145 (14)	−0.0001 (12)	−0.0026 (11)	−0.0020 (11)
C37	0.0164 (14)	0.0140 (14)	0.0101 (12)	−0.0028 (12)	−0.0042 (11)	0.0018 (10)
C38	0.0179 (15)	0.0128 (14)	0.0158 (13)	−0.0057 (12)	−0.0040 (11)	0.0013 (11)
C39	0.0211 (15)	0.0138 (14)	0.0138 (13)	−0.0026 (12)	−0.0015 (11)	0.0000 (11)
C40	0.0172 (15)	0.0222 (16)	0.0148 (14)	−0.0018 (13)	0.0010 (11)	0.0037 (12)
C41	0.0211 (16)	0.0272 (17)	0.0245 (16)	−0.0139 (14)	−0.0046 (13)	0.0037 (13)
C42	0.0234 (16)	0.0152 (15)	0.0211 (15)	−0.0073 (13)	−0.0033 (12)	0.0000 (12)
Ni2	0.01326 (18)	0.01057 (17)	0.01248 (17)	−0.00355 (14)	−0.00116 (13)	−0.00184 (13)
F5	0.0703 (17)	0.0144 (10)	0.0599 (15)	−0.0068 (11)	0.0186 (13)	0.0064 (10)
F6	0.0318 (13)	0.113 (2)	0.0501 (15)	−0.0329 (15)	−0.0135 (11)	0.0024 (15)
F7	0.0231 (10)	0.0191 (9)	0.0266 (10)	−0.0016 (8)	−0.0026 (8)	−0.0071 (7)
F8	0.0427 (14)	0.0562 (15)	0.0556 (15)	−0.0294 (12)	−0.0054 (11)	0.0227 (12)
N7	0.0141 (12)	0.0123 (12)	0.0183 (12)	−0.0018 (10)	−0.0053 (10)	−0.0020 (9)
N8	0.0166 (12)	0.0126 (12)	0.0154 (12)	−0.0008 (10)	−0.0015 (10)	0.0001 (9)
N9	0.0142 (12)	0.0225 (14)	0.0146 (12)	−0.0017 (11)	−0.0017 (10)	−0.0032 (10)
N10	0.0178 (13)	0.0165 (12)	0.0101 (11)	−0.0068 (10)	0.0024 (9)	−0.0023 (9)
N11	0.0143 (12)	0.0162 (13)	0.0148 (12)	−0.0034 (10)	−0.0009 (9)	−0.0016 (9)
N12	0.0168 (13)	0.0139 (12)	0.0123 (11)	−0.0022 (10)	0.0001 (9)	−0.0031 (9)
C43	0.0166 (15)	0.0129 (14)	0.0218 (15)	−0.0046 (12)	−0.0073 (12)	0.0021 (11)
C44	0.0253 (17)	0.0190 (16)	0.0309 (17)	−0.0101 (14)	−0.0088 (14)	0.0048 (13)
C45	0.037 (2)	0.0155 (16)	0.040 (2)	−0.0112 (15)	−0.0162 (16)	−0.0007 (14)
C46	0.0306 (19)	0.0171 (16)	0.0339 (18)	−0.0017 (14)	−0.0125 (15)	−0.0081 (14)
C47	0.0191 (15)	0.0132 (14)	0.0215 (15)	0.0012 (12)	−0.0078 (12)	−0.0047 (12)
C48	0.0153 (15)	0.0179 (15)	0.0196 (15)	−0.0051 (12)	−0.0013 (12)	0.0050 (12)
C49	0.0274 (17)	0.0157 (15)	0.0173 (15)	−0.0001 (13)	0.0019 (13)	−0.0029 (12)
C50	0.0178 (15)	0.0146 (14)	0.0158 (14)	−0.0028 (12)	0.0043 (11)	−0.0040 (11)
C51	0.0260 (17)	0.0186 (16)	0.0302 (17)	−0.0087 (14)	−0.0063 (14)	−0.0015 (13)
C52	0.033 (2)	0.0245 (18)	0.0323 (19)	0.0010 (15)	−0.0064 (15)	0.0031 (15)
C53	0.044 (2)	0.0145 (16)	0.0324 (19)	−0.0056 (15)	0.0144 (16)	0.0002 (14)
C54	0.0315 (19)	0.0246 (18)	0.041 (2)	−0.0162 (16)	0.0143 (16)	−0.0119 (15)
C55	0.0178 (16)	0.0279 (18)	0.0256 (16)	−0.0051 (14)	0.0042 (13)	−0.0110 (14)
C56	0.0169 (15)	0.0221 (16)	0.0187 (15)	0.0034 (13)	−0.0047 (12)	−0.0103 (12)
C57	0.0216 (16)	0.0331 (19)	0.0155 (14)	−0.0058 (14)	0.0030 (12)	−0.0029 (13)
C58	0.0190 (16)	0.037 (2)	0.0186 (15)	−0.0063 (15)	0.0056 (12)	−0.0068 (14)
C59	0.0209 (17)	0.041 (2)	0.0236 (17)	−0.0030 (15)	0.0039 (13)	−0.0025 (15)
C60	0.0192 (18)	0.063 (3)	0.0251 (18)	−0.0037 (18)	−0.0006 (14)	0.0057 (18)
C61	0.0206 (18)	0.075 (3)	0.0293 (19)	−0.014 (2)	−0.0049 (15)	−0.0025 (19)
C62	0.029 (2)	0.057 (3)	0.048 (2)	−0.022 (2)	0.0016 (18)	−0.001 (2)
C63	0.0255 (19)	0.043 (2)	0.0321 (19)	−0.0086 (17)	−0.0002 (15)	0.0046 (16)
C64	0.0181 (15)	0.0224 (16)	0.0120 (13)	−0.0100 (13)	0.0025 (11)	−0.0040 (11)
C65	0.0266 (17)	0.0274 (17)	0.0193 (15)	−0.0175 (14)	0.0018 (13)	−0.0042 (13)
C66	0.039 (2)	0.0232 (17)	0.0261 (17)	−0.0221 (16)	0.0051 (15)	−0.0023 (13)
C67	0.0351 (19)	0.0207 (16)	0.0173 (15)	−0.0134 (14)	0.0036 (13)	0.0003 (12)
C68	0.0239 (16)	0.0171 (15)	0.0106 (13)	−0.0083 (13)	0.0038 (11)	−0.0013 (11)
C69	0.0196 (15)	0.0150 (15)	0.0135 (13)	−0.0005 (12)	0.0008 (11)	0.0004 (11)
C70	0.0174 (15)	0.0176 (15)	0.0168 (14)	−0.0013 (12)	−0.0052 (12)	−0.0026 (11)
C71	0.0160 (14)	0.0174 (15)	0.0142 (13)	−0.0030 (12)	−0.0063 (11)	0.0012 (11)
C72	0.0171 (15)	0.0241 (16)	0.0167 (14)	−0.0077 (13)	−0.0005 (12)	−0.0019 (12)
C73	0.0207 (16)	0.0199 (16)	0.0206 (15)	−0.0096 (13)	−0.0053 (12)	−0.0003 (12)
C74	0.0169 (15)	0.0150 (14)	0.0175 (14)	0.0003 (12)	−0.0063 (11)	−0.0038 (11)
C75	0.0164 (15)	0.0215 (16)	0.0144 (14)	−0.0045 (12)	−0.0016 (11)	0.0005 (12)
C76	0.0184 (15)	0.0182 (15)	0.0154 (14)	−0.0062 (12)	−0.0055 (11)	0.0040 (11)
C77	0.0153 (15)	0.0236 (16)	0.0135 (13)	−0.0060 (13)	−0.0002 (11)	−0.0034 (12)
C78	0.0174 (15)	0.0161 (15)	0.0215 (15)	0.0010 (12)	−0.0049 (12)	0.0020 (12)
C79	0.0188 (15)	0.0134 (14)	0.0196 (15)	−0.0008 (12)	−0.0064 (12)	0.0036 (11)
C80	0.0282 (18)	0.0151 (15)	0.0242 (16)	−0.0040 (13)	−0.0067 (13)	0.0043 (12)
C81	0.0280 (18)	0.0241 (18)	0.0284 (18)	−0.0008 (15)	0.0023 (14)	0.0072 (14)
C82	0.0291 (19)	0.035 (2)	0.0340 (19)	−0.0171 (16)	−0.0071 (15)	0.0160 (16)
C83	0.044 (2)	0.0218 (18)	0.0353 (19)	−0.0183 (16)	−0.0173 (17)	0.0092 (15)
C84	0.0327 (19)	0.0170 (16)	0.0243 (16)	−0.0042 (14)	−0.0109 (14)	0.0017 (13)
Br1	0.01836 (15)	0.01624 (15)	0.02801 (16)	−0.00542 (12)	0.00252 (12)	−0.00338 (12)
Br2	0.03089 (18)	0.02165 (17)	0.02241 (16)	−0.00779 (14)	−0.00133 (13)	−0.00384 (12)
Br3	0.02149 (17)	0.03430 (19)	0.02683 (17)	−0.00630 (14)	0.00480 (13)	0.00590 (14)
Br4	0.02946 (18)	0.02484 (17)	0.03397 (18)	−0.01384 (14)	−0.00543 (14)	−0.00575 (14)
O1	0.0340 (15)	0.0261 (13)	0.0441 (15)	−0.0103 (12)	−0.0014 (12)	0.0001 (11)
O2	0.0323 (14)	0.0226 (13)	0.0427 (15)	−0.0036 (11)	−0.0120 (12)	−0.0086 (11)
O3	0.0502 (17)	0.0197 (12)	0.0278 (13)	−0.0075 (12)	0.0080 (12)	−0.0044 (10)
O4	0.0369 (16)	0.0457 (18)	0.0515 (18)	0.0006 (14)	−0.0119 (14)	0.0031 (14)
O5	0.0388 (17)	0.054 (2)	0.0511 (19)	−0.0050 (15)	0.0007 (14)	0.0132 (15)
O6	0.0307 (14)	0.0213 (13)	0.0418 (15)	−0.0063 (11)	0.0031 (12)	−0.0035 (11)
O7	0.0322 (15)	0.0289 (14)	0.0532 (17)	−0.0109 (12)	−0.0159 (13)	−0.0025 (12)
O8	0.088 (3)	0.065 (2)	0.0453 (19)	−0.026 (2)	0.0054 (18)	0.0024 (16)
O9	0.070 (3)	0.100 (3)	0.059 (2)	−0.022 (2)	−0.0060 (19)	−0.002 (2)

### Geometric parameters (Å, º)

**Table:**

Ni1—N4	1.984 (2)	F8—C82	1.366 (4)
Ni1—N1	1.984 (2)	N7—C47	1.333 (4)
Ni1—N6	2.141 (2)	N7—C43	1.339 (4)
Ni1—N5	2.151 (2)	N8—C48	1.280 (4)
Ni1—N2	2.158 (2)	N8—C49	1.474 (4)
Ni1—N3	2.165 (2)	N9—C56	1.277 (4)
F1—C11	1.369 (4)	N9—C57	1.469 (4)
F2—C19	1.378 (4)	N10—C68	1.340 (4)
F3—C32	1.368 (4)	N10—C64	1.342 (4)
F4—C40	1.362 (3)	N11—C77	1.278 (4)
N1—C1	1.335 (4)	N11—C78	1.469 (4)
N1—C5	1.347 (4)	N12—C69	1.276 (4)
N2—C6	1.281 (4)	N12—C70	1.471 (4)
N2—C7	1.469 (4)	C43—C44	1.388 (4)
N3—C14	1.267 (4)	C43—C48	1.469 (4)
N3—C15	1.472 (4)	C44—C45	1.393 (5)
N4—C22	1.336 (4)	C44—H44	0.9500
N4—C26	1.341 (4)	C45—C46	1.377 (5)
N5—C27	1.278 (4)	C45—H45	0.9500
N5—C28	1.469 (3)	C46—C47	1.396 (4)
N6—C35	1.275 (4)	C46—H46	0.9500
N6—C36	1.470 (4)	C47—C56	1.470 (4)
C1—C2	1.390 (4)	C48—H48	0.9500
C1—C6	1.473 (4)	C49—C50	1.503 (4)
C2—C3	1.391 (5)	C49—H491	0.9900
C2—H2	0.9500	C49—H492	0.9900
C3—C4	1.394 (5)	C50—C55	1.390 (4)
C3—H3	0.9500	C50—C51	1.393 (4)
C4—C5	1.384 (4)	C51—C52	1.387 (5)
C4—H4	0.9500	C51—H51	0.9500
C5—C14	1.479 (4)	C52—C53	1.374 (5)
C6—H6	0.9500	C52—H52	0.9500
C7—C8	1.511 (4)	C53—C54	1.363 (5)
C7—H71	0.9900	C54—C55	1.383 (5)
C7—H72	0.9900	C54—H54	0.9500
C8—C13	1.388 (4)	C55—H55	0.9500
C8—C9	1.389 (4)	C56—H56	0.9500
C9—C10	1.395 (4)	C57—C58	1.514 (5)
C9—H9	0.9500	C57—H571	0.9900
C10—C11	1.372 (5)	C57—H572	0.9900
C10—H10	0.9500	C58—C63	1.384 (5)
C11—C12	1.378 (5)	C58—C59	1.391 (5)
C12—C13	1.395 (4)	C59—C60	1.382 (5)
C12—H12	0.9500	C59—H59	0.9500
C13—H13	0.9500	C60—C61	1.363 (6)
C14—H14	0.9500	C60—H60	0.9500
C15—C16	1.499 (4)	C61—C62	1.364 (6)
C15—H151	0.9900	C62—C63	1.405 (5)
C15—H152	0.9900	C62—H62	0.9500
C16—C17	1.387 (4)	C63—H63	0.9500
C16—C21	1.389 (4)	C64—C65	1.387 (4)
C17—C18	1.415 (5)	C64—C77	1.472 (4)
C17—H17	0.9500	C65—C66	1.386 (5)
C18—C19	1.364 (5)	C65—H65	0.9500
C18—H18	0.9500	C66—C67	1.387 (5)
C19—C20	1.359 (5)	C66—H66	0.9500
C20—C21	1.383 (4)	C67—C68	1.392 (4)
C20—H20	0.9500	C67—H67	0.9500
C21—H21	0.9500	C68—C69	1.471 (4)
C22—C23	1.393 (4)	C69—H69	0.9500
C22—C27	1.474 (4)	C70—C71	1.513 (4)
C23—C24	1.387 (4)	C70—H701	0.9900
C23—H23	0.9500	C70—H702	0.9900
C24—C25	1.388 (4)	C71—C76	1.387 (4)
C24—H24	0.9500	C71—C72	1.395 (4)
C25—C26	1.387 (4)	C72—C73	1.391 (4)
C25—H25	0.9500	C72—H721	0.9500
C26—C35	1.469 (4)	C73—C74	1.370 (4)
C27—H27	0.9500	C73—H73	0.9500
C28—C29	1.509 (4)	C74—C75	1.380 (4)
C28—H281	0.9900	C75—C76	1.394 (4)
C28—H282	0.9900	C75—H75	0.9500
C29—C30	1.387 (4)	C76—H76	0.9500
C29—C34	1.395 (4)	C77—H77	0.9500
C30—C31	1.391 (5)	C78—C79	1.512 (4)
C30—H30	0.9500	C78—H781	0.9900
C31—C32	1.375 (6)	C78—H782	0.9900
C31—H31	0.9500	C79—C80	1.388 (4)
C32—C33	1.364 (5)	C79—C84	1.390 (4)
C33—C34	1.389 (4)	C80—C81	1.384 (5)
C33—H33	0.9500	C80—H80	0.9500
C34—H34	0.9500	C81—C82	1.385 (5)
C35—H35	0.9500	C81—H81	0.9500
C36—C37	1.516 (4)	C82—C83	1.358 (5)
C36—H361	0.9900	C83—C84	1.387 (5)
C36—H362	0.9900	C83—H83	0.9500
C37—C42	1.393 (4)	C84—H84	0.9500
C37—C38	1.400 (4)	O1—H111	0.893 (18)
C38—C39	1.388 (4)	O1—H112	0.878 (18)
C38—H38	0.9500	O2—H211	0.893 (18)
C39—C40	1.373 (4)	O2—H212	0.873 (18)
C39—H39	0.9500	O3—H311	0.899 (18)
C40—C41	1.375 (5)	O3—H312	0.892 (18)
C41—C42	1.391 (4)	O4—H411	0.949 (19)
C41—H41	0.9500	O4—H412	0.933 (19)
C42—H42	0.9500	O5—H511	0.884 (19)
Ni2—N10	1.973 (2)	O5—H512	0.896 (19)
Ni2—N7	1.981 (2)	O6—H611	0.863 (18)
Ni2—N11	2.125 (2)	O6—H612	0.868 (18)
Ni2—N9	2.158 (2)	O7—H711	0.899 (18)
Ni2—N12	2.161 (2)	O7—H712	0.876 (18)
Ni2—N8	2.169 (2)	O8—H811	0.936 (19)
F5—C53	1.369 (4)	O8—H812	0.943 (19)
F6—C61	1.373 (4)	O9—H911	0.971 (19)
F7—C74	1.367 (3)	O9—H912	0.967 (19)
			
N4—Ni1—N1	176.85 (10)	N11—Ni2—N9	89.68 (9)
N4—Ni1—N6	77.31 (9)	N10—Ni2—N12	77.22 (10)
N1—Ni1—N6	100.97 (9)	N7—Ni2—N12	102.95 (9)
N4—Ni1—N5	77.05 (9)	N11—Ni2—N12	154.80 (9)
N1—Ni1—N5	104.62 (9)	N9—Ni2—N12	94.39 (9)
N6—Ni1—N5	154.36 (9)	N10—Ni2—N8	105.89 (9)
N4—Ni1—N2	100.20 (9)	N7—Ni2—N8	77.01 (10)
N1—Ni1—N2	77.09 (9)	N11—Ni2—N8	95.48 (9)
N6—Ni1—N2	90.34 (9)	N9—Ni2—N8	154.21 (10)
N5—Ni1—N2	93.75 (9)	N12—Ni2—N8	91.60 (9)
N4—Ni1—N3	105.70 (9)	C47—N7—C43	121.1 (3)
N1—Ni1—N3	76.99 (9)	C47—N7—Ni2	119.3 (2)
N6—Ni1—N3	94.22 (9)	C43—N7—Ni2	119.7 (2)
N5—Ni1—N3	93.06 (9)	C48—N8—C49	118.3 (3)
N2—Ni1—N3	154.08 (9)	C48—N8—Ni2	112.4 (2)
C1—N1—C5	120.5 (2)	C49—N8—Ni2	129.1 (2)
C1—N1—Ni1	119.77 (19)	C56—N9—C57	118.4 (3)
C5—N1—Ni1	119.69 (19)	C56—N9—Ni2	112.6 (2)
C6—N2—C7	117.6 (2)	C57—N9—Ni2	128.9 (2)
C6—N2—Ni1	112.71 (19)	C68—N10—C64	121.1 (3)
C7—N2—Ni1	129.34 (19)	C68—N10—Ni2	119.6 (2)
C14—N3—C15	117.8 (3)	C64—N10—Ni2	119.2 (2)
C14—N3—Ni1	112.9 (2)	C77—N11—C78	118.5 (3)
C15—N3—Ni1	129.1 (2)	C77—N11—Ni2	113.6 (2)
C22—N4—C26	121.1 (2)	C78—N11—Ni2	127.60 (19)
C22—N4—Ni1	119.76 (19)	C69—N12—C70	118.7 (2)
C26—N4—Ni1	119.12 (19)	C69—N12—Ni2	112.6 (2)
C27—N5—C28	118.4 (2)	C70—N12—Ni2	128.62 (18)
C27—N5—Ni1	113.13 (19)	N7—C43—C44	120.9 (3)
C28—N5—Ni1	128.45 (19)	N7—C43—C48	112.5 (2)
C35—N6—C36	118.6 (2)	C44—C43—C48	126.6 (3)
C35—N6—Ni1	113.24 (19)	C43—C44—C45	118.2 (3)
C36—N6—Ni1	128.19 (18)	C43—C44—H44	120.9
N1—C1—C2	121.5 (3)	C45—C44—H44	120.9
N1—C1—C6	112.4 (2)	C46—C45—C44	120.5 (3)
C2—C1—C6	126.0 (3)	C46—C45—H45	119.7
C1—C2—C3	118.4 (3)	C44—C45—H45	119.7
C1—C2—H2	120.8	C45—C46—C47	118.0 (3)
C3—C2—H2	120.8	C45—C46—H46	121.0
C2—C3—C4	119.8 (3)	C47—C46—H46	121.0
C2—C3—H3	120.1	N7—C47—C46	121.3 (3)
C4—C3—H3	120.1	N7—C47—C56	112.9 (3)
C5—C4—C3	118.5 (3)	C46—C47—C56	125.7 (3)
C5—C4—H4	120.8	N8—C48—C43	118.1 (3)
C3—C4—H4	120.8	N8—C48—H48	121.0
N1—C5—C4	121.4 (3)	C43—C48—H48	121.0
N1—C5—C14	111.8 (2)	N8—C49—C50	112.4 (2)
C4—C5—C14	126.8 (3)	N8—C49—H491	109.1
N2—C6—C1	118.0 (3)	C50—C49—H491	109.1
N2—C6—H6	121.0	N8—C49—H492	109.1
C1—C6—H6	121.0	C50—C49—H492	109.1
N2—C7—C8	113.1 (2)	H491—C49—H492	107.8
N2—C7—H71	109.0	C55—C50—C51	118.6 (3)
C8—C7—H71	109.0	C55—C50—C49	120.4 (3)
N2—C7—H72	109.0	C51—C50—C49	121.0 (3)
C8—C7—H72	109.0	C52—C51—C50	120.9 (3)
H71—C7—H72	107.8	C52—C51—H51	119.6
C13—C8—C9	119.4 (3)	C50—C51—H51	119.6
C13—C8—C7	121.3 (3)	C53—C52—C51	117.8 (3)
C9—C8—C7	119.3 (3)	C53—C52—H52	121.1
C8—C9—C10	121.0 (3)	C51—C52—H52	121.1
C8—C9—H9	119.5	C54—C53—F5	118.8 (3)
C10—C9—H9	119.5	C54—C53—C52	123.5 (3)
C11—C10—C9	117.6 (3)	F5—C53—C52	117.7 (3)
C11—C10—H10	121.2	C53—C54—C55	117.9 (3)
C9—C10—H10	121.2	C53—C54—H54	121.0
F1—C11—C10	118.1 (3)	C55—C54—H54	121.0
F1—C11—C12	118.4 (3)	C54—C55—C50	121.3 (3)
C10—C11—C12	123.5 (3)	C54—C55—H55	119.3
C11—C12—C13	117.8 (3)	C50—C55—H55	119.3
C11—C12—H12	121.1	N9—C56—C47	117.7 (3)
C13—C12—H12	121.1	N9—C56—H56	121.1
C8—C13—C12	120.7 (3)	C47—C56—H56	121.1
C8—C13—H13	119.7	N9—C57—C58	112.8 (2)
C12—C13—H13	119.7	N9—C57—H571	109.0
N3—C14—C5	118.4 (3)	C58—C57—H571	109.0
N3—C14—H14	120.8	N9—C57—H572	109.0
C5—C14—H14	120.8	C58—C57—H572	109.0
N3—C15—C16	113.2 (2)	H571—C57—H572	107.8
N3—C15—H151	108.9	C63—C58—C59	119.2 (3)
C16—C15—H151	108.9	C63—C58—C57	119.5 (3)
N3—C15—H152	108.9	C59—C58—C57	121.2 (3)
C16—C15—H152	108.9	C60—C59—C58	120.9 (4)
H151—C15—H152	107.7	C60—C59—H59	119.6
C17—C16—C21	119.2 (3)	C58—C59—H59	119.6
C17—C16—C15	121.7 (3)	C61—C60—C59	118.1 (4)
C21—C16—C15	119.2 (3)	C61—C60—H60	121.0
C16—C17—C18	119.4 (3)	C59—C60—H60	121.0
C16—C17—H17	120.3	C60—C61—C62	123.9 (4)
C18—C17—H17	120.3	C60—C61—F6	118.7 (4)
C19—C18—C17	118.2 (3)	C62—C61—F6	117.5 (4)
C19—C18—H18	120.9	C61—C62—C63	117.5 (4)
C17—C18—H18	120.9	C61—C62—H62	121.2
C20—C19—C18	124.1 (3)	C63—C62—H62	121.2
C20—C19—F2	117.6 (3)	C58—C63—C62	120.5 (4)
C18—C19—F2	118.3 (3)	C58—C63—H63	119.8
C19—C20—C21	117.2 (3)	C62—C63—H63	119.8
C19—C20—H20	121.4	N10—C64—C65	121.1 (3)
C21—C20—H20	121.4	N10—C64—C77	112.3 (3)
C20—C21—C16	122.0 (3)	C65—C64—C77	126.5 (3)
C20—C21—H21	119.0	C66—C65—C64	118.0 (3)
C16—C21—H21	119.0	C66—C65—H65	121.0
N4—C22—C23	121.3 (3)	C64—C65—H65	121.0
N4—C22—C27	112.3 (2)	C65—C66—C67	120.8 (3)
C23—C22—C27	126.4 (3)	C65—C66—H66	119.6
C24—C23—C22	117.9 (3)	C67—C66—H66	119.6
C24—C23—H23	121.1	C66—C67—C68	118.0 (3)
C22—C23—H23	121.1	C66—C67—H67	121.0
C23—C24—C25	120.3 (3)	C68—C67—H67	121.0
C23—C24—H24	119.8	N10—C68—C67	120.9 (3)
C25—C24—H24	119.8	N10—C68—C69	112.5 (2)
C26—C25—C24	118.6 (3)	C67—C68—C69	126.6 (3)
C26—C25—H25	120.7	N12—C69—C68	117.9 (3)
C24—C25—H25	120.7	N12—C69—H69	121.0
N4—C26—C25	120.7 (3)	C68—C69—H69	121.0
N4—C26—C35	112.4 (2)	N12—C70—C71	110.2 (2)
C25—C26—C35	126.8 (3)	N12—C70—H701	109.6
N5—C27—C22	117.8 (3)	C71—C70—H701	109.6
N5—C27—H27	121.1	N12—C70—H702	109.6
C22—C27—H27	121.1	C71—C70—H702	109.6
N5—C28—C29	110.3 (2)	H701—C70—H702	108.1
N5—C28—H281	109.6	C76—C71—C72	119.5 (3)
C29—C28—H281	109.6	C76—C71—C70	119.8 (3)
N5—C28—H282	109.6	C72—C71—C70	120.7 (3)
C29—C28—H282	109.6	C73—C72—C71	120.4 (3)
H281—C28—H282	108.1	C73—C72—H721	119.8
C30—C29—C34	119.1 (3)	C71—C72—H721	119.8
C30—C29—C28	121.2 (3)	C74—C73—C72	118.1 (3)
C34—C29—C28	119.7 (3)	C74—C73—H73	120.9
C29—C30—C31	120.4 (3)	C72—C73—H73	120.9
C29—C30—H30	119.8	F7—C74—C73	118.3 (3)
C31—C30—H30	119.8	F7—C74—C75	118.1 (3)
C32—C31—C30	118.4 (3)	C73—C74—C75	123.5 (3)
C32—C31—H31	120.8	C74—C75—C76	117.5 (3)
C30—C31—H31	120.8	C74—C75—H75	121.3
C33—C32—F3	118.1 (3)	C76—C75—H75	121.3
C33—C32—C31	123.3 (3)	C71—C76—C75	120.9 (3)
F3—C32—C31	118.6 (3)	C71—C76—H76	119.6
C32—C33—C34	117.8 (3)	C75—C76—H76	119.6
C32—C33—H33	121.1	N11—C77—C64	117.3 (3)
C34—C33—H33	121.1	N11—C77—H77	121.3
C33—C34—C29	121.1 (3)	C64—C77—H77	121.3
C33—C34—H34	119.5	N11—C78—C79	111.6 (2)
C29—C34—H34	119.5	N11—C78—H781	109.3
N6—C35—C26	117.9 (3)	C79—C78—H781	109.3
N6—C35—H35	121.1	N11—C78—H782	109.3
C26—C35—H35	121.1	C79—C78—H782	109.3
N6—C36—C37	110.8 (2)	H781—C78—H782	108.0
N6—C36—H361	109.5	C80—C79—C84	119.3 (3)
C37—C36—H361	109.5	C80—C79—C78	120.4 (3)
N6—C36—H362	109.5	C84—C79—C78	120.2 (3)
C37—C36—H362	109.5	C81—C80—C79	120.7 (3)
H361—C36—H362	108.1	C81—C80—H80	119.7
C42—C37—C38	118.8 (3)	C79—C80—H80	119.7
C42—C37—C36	121.2 (3)	C80—C81—C82	117.9 (3)
C38—C37—C36	119.9 (3)	C80—C81—H81	121.1
C39—C38—C37	121.1 (3)	C82—C81—H81	121.1
C39—C38—H38	119.5	C83—C82—F8	118.9 (3)
C37—C38—H38	119.5	C83—C82—C81	123.1 (3)
C40—C39—C38	118.1 (3)	F8—C82—C81	117.9 (3)
C40—C39—H39	120.9	C82—C83—C84	118.3 (3)
C38—C39—H39	120.9	C82—C83—H83	120.8
F4—C40—C39	118.6 (3)	C84—C83—H83	120.8
F4—C40—C41	118.7 (3)	C83—C84—C79	120.6 (3)
C39—C40—C41	122.7 (3)	C83—C84—H84	119.7
C40—C41—C42	118.9 (3)	C79—C84—H84	119.7
C40—C41—H41	120.6	H111—O1—H112	106 (3)
C42—C41—H41	120.6	H211—O2—H212	102 (3)
C41—C42—C37	120.3 (3)	H311—O3—H312	100 (3)
C41—C42—H42	119.8	H411—O4—H412	95 (3)
C37—C42—H42	119.8	H511—O5—H512	100 (3)
N10—Ni2—N7	177.10 (10)	H611—O6—H612	108 (3)
N10—Ni2—N11	77.58 (10)	H711—O7—H712	92 (3)
N7—Ni2—N11	102.20 (9)	H811—O8—H812	84 (3)
N10—Ni2—N9	99.90 (10)	H911—O9—H912	96 (3)
N7—Ni2—N9	77.20 (10)		
			
C5—N1—C1—C2	−0.5 (4)	C47—N7—C43—C44	−1.9 (4)
Ni1—N1—C1—C2	−177.8 (2)	Ni2—N7—C43—C44	177.0 (2)
C5—N1—C1—C6	175.8 (2)	C47—N7—C43—C48	176.1 (3)
Ni1—N1—C1—C6	−1.5 (3)	Ni2—N7—C43—C48	−4.9 (3)
N1—C1—C2—C3	0.9 (4)	N7—C43—C44—C45	2.5 (4)
C6—C1—C2—C3	−174.9 (3)	C48—C43—C44—C45	−175.3 (3)
C1—C2—C3—C4	−0.4 (4)	C43—C44—C45—C46	−0.3 (5)
C2—C3—C4—C5	−0.4 (4)	C44—C45—C46—C47	−2.3 (5)
C1—N1—C5—C4	−0.4 (4)	C43—N7—C47—C46	−0.8 (4)
Ni1—N1—C5—C4	177.0 (2)	Ni2—N7—C47—C46	−179.8 (2)
C1—N1—C5—C14	178.4 (2)	C43—N7—C47—C56	176.4 (3)
Ni1—N1—C5—C14	−4.3 (3)	Ni2—N7—C47—C56	−2.6 (3)
C3—C4—C5—N1	0.8 (4)	C45—C46—C47—N7	2.9 (5)
C3—C4—C5—C14	−177.7 (3)	C45—C46—C47—C56	−173.9 (3)
C7—N2—C6—C1	−173.0 (2)	C49—N8—C48—C43	−172.5 (2)
Ni1—N2—C6—C1	1.1 (3)	Ni2—N8—C48—C43	2.7 (3)
N1—C1—C6—N2	0.1 (4)	N7—C43—C48—N8	1.1 (4)
C2—C1—C6—N2	176.2 (3)	C44—C43—C48—N8	179.0 (3)
C6—N2—C7—C8	−128.3 (3)	C48—N8—C49—C50	−129.7 (3)
Ni1—N2—C7—C8	58.7 (3)	Ni2—N8—C49—C50	56.0 (3)
N2—C7—C8—C13	51.3 (4)	N8—C49—C50—C55	−112.5 (3)
N2—C7—C8—C9	−130.6 (3)	N8—C49—C50—C51	69.2 (4)
C13—C8—C9—C10	0.4 (4)	C55—C50—C51—C52	1.1 (5)
C7—C8—C9—C10	−177.7 (3)	C49—C50—C51—C52	179.5 (3)
C8—C9—C10—C11	−2.2 (5)	C50—C51—C52—C53	−0.7 (5)
C9—C10—C11—F1	−178.4 (3)	C51—C52—C53—C54	−0.3 (5)
C9—C10—C11—C12	2.5 (5)	C51—C52—C53—F5	−179.9 (3)
F1—C11—C12—C13	179.8 (3)	F5—C53—C54—C55	−179.5 (3)
C10—C11—C12—C13	−1.0 (5)	C52—C53—C54—C55	0.9 (5)
C9—C8—C13—C12	1.1 (4)	C53—C54—C55—C50	−0.5 (5)
C7—C8—C13—C12	179.2 (3)	C51—C50—C55—C54	−0.5 (5)
C11—C12—C13—C8	−0.8 (5)	C49—C50—C55—C54	−178.9 (3)
C15—N3—C14—C5	−175.9 (2)	C57—N9—C56—C47	−173.5 (3)
Ni1—N3—C14—C5	1.4 (3)	Ni2—N9—C56—C47	4.1 (3)
N1—C5—C14—N3	1.7 (4)	N7—C47—C56—N9	−1.3 (4)
C4—C5—C14—N3	−179.7 (3)	C46—C47—C56—N9	175.7 (3)
C14—N3—C15—C16	−131.9 (3)	C56—N9—C57—C58	−114.9 (3)
Ni1—N3—C15—C16	51.4 (4)	Ni2—N9—C57—C58	67.9 (3)
N3—C15—C16—C17	61.3 (4)	N9—C57—C58—C63	−132.6 (3)
N3—C15—C16—C21	−120.1 (3)	N9—C57—C58—C59	50.2 (4)
C21—C16—C17—C18	0.1 (5)	C63—C58—C59—C60	−0.2 (5)
C15—C16—C17—C18	178.7 (3)	C57—C58—C59—C60	177.0 (3)
C16—C17—C18—C19	0.2 (5)	C58—C59—C60—C61	−0.7 (5)
C17—C18—C19—C20	−1.2 (6)	C59—C60—C61—C62	0.8 (6)
C17—C18—C19—F2	178.0 (3)	C59—C60—C61—F6	−178.2 (3)
C18—C19—C20—C21	1.8 (5)	C60—C61—C62—C63	0.0 (6)
F2—C19—C20—C21	−177.3 (3)	F6—C61—C62—C63	179.0 (3)
C19—C20—C21—C16	−1.5 (5)	C59—C58—C63—C62	1.0 (5)
C17—C16—C21—C20	0.6 (5)	C57—C58—C63—C62	−176.2 (3)
C15—C16—C21—C20	−178.1 (3)	C61—C62—C63—C58	−1.0 (6)
C26—N4—C22—C23	1.0 (4)	C68—N10—C64—C65	1.1 (4)
Ni1—N4—C22—C23	178.4 (2)	Ni2—N10—C64—C65	177.0 (2)
C26—N4—C22—C27	−177.5 (2)	C68—N10—C64—C77	−176.7 (2)
Ni1—N4—C22—C27	−0.1 (3)	Ni2—N10—C64—C77	−0.8 (3)
N4—C22—C23—C24	−0.6 (4)	N10—C64—C65—C66	−1.2 (4)
C27—C22—C23—C24	177.7 (3)	C77—C64—C65—C66	176.2 (3)
C22—C23—C24—C25	−0.2 (4)	C64—C65—C66—C67	0.3 (5)
C23—C24—C25—C26	0.7 (4)	C65—C66—C67—C68	0.6 (5)
C22—N4—C26—C25	−0.5 (4)	C64—N10—C68—C67	−0.1 (4)
Ni1—N4—C26—C25	−177.9 (2)	Ni2—N10—C68—C67	−176.0 (2)
C22—N4—C26—C35	179.2 (2)	C64—N10—C68—C69	179.6 (2)
Ni1—N4—C26—C35	1.8 (3)	Ni2—N10—C68—C69	3.8 (3)
C24—C25—C26—N4	−0.4 (4)	C66—C67—C68—N10	−0.7 (4)
C24—C25—C26—C35	−180.0 (3)	C66—C67—C68—C69	179.6 (3)
C28—N5—C27—C22	179.5 (2)	C70—N12—C69—C68	177.8 (2)
Ni1—N5—C27—C22	1.9 (3)	Ni2—N12—C69—C68	−0.9 (3)
N4—C22—C27—N5	−1.3 (4)	N10—C68—C69—N12	−1.7 (4)
C23—C22—C27—N5	−179.7 (3)	C67—C68—C69—N12	178.1 (3)
C27—N5—C28—C29	115.7 (3)	C69—N12—C70—C71	123.2 (3)
Ni1—N5—C28—C29	−67.1 (3)	Ni2—N12—C70—C71	−58.3 (3)
N5—C28—C29—C30	116.8 (3)	N12—C70—C71—C76	−59.2 (3)
N5—C28—C29—C34	−60.3 (3)	N12—C70—C71—C72	121.5 (3)
C34—C29—C30—C31	0.3 (5)	C76—C71—C72—C73	1.8 (4)
C28—C29—C30—C31	−176.8 (3)	C70—C71—C72—C73	−178.9 (3)
C29—C30—C31—C32	0.2 (5)	C71—C72—C73—C74	−1.0 (4)
C30—C31—C32—C33	−0.9 (6)	C72—C73—C74—F7	179.6 (3)
C30—C31—C32—F3	179.6 (3)	C72—C73—C74—C75	0.0 (5)
F3—C32—C33—C34	−179.5 (3)	F7—C74—C75—C76	−179.4 (2)
C31—C32—C33—C34	1.1 (5)	C73—C74—C75—C76	0.2 (4)
C32—C33—C34—C29	−0.6 (5)	C72—C71—C76—C75	−1.6 (4)
C30—C29—C34—C33	−0.1 (4)	C70—C71—C76—C75	179.0 (3)
C28—C29—C34—C33	177.0 (3)	C74—C75—C76—C71	0.6 (4)
C36—N6—C35—C26	179.4 (2)	C78—N11—C77—C64	173.7 (2)
Ni1—N6—C35—C26	0.3 (3)	Ni2—N11—C77—C64	−0.2 (3)
N4—C26—C35—N6	−1.3 (4)	N10—C64—C77—N11	0.7 (4)
C25—C26—C35—N6	178.3 (3)	C65—C64—C77—N11	−177.0 (3)
C35—N6—C36—C37	106.9 (3)	C77—N11—C78—C79	128.7 (3)
Ni1—N6—C36—C37	−74.2 (3)	Ni2—N11—C78—C79	−58.3 (3)
N6—C36—C37—C42	129.2 (3)	N11—C78—C79—C80	−55.9 (4)
N6—C36—C37—C38	−53.9 (3)	N11—C78—C79—C84	125.7 (3)
C42—C37—C38—C39	−2.0 (4)	C84—C79—C80—C81	−3.0 (5)
C36—C37—C38—C39	−178.9 (2)	C78—C79—C80—C81	178.6 (3)
C37—C38—C39—C40	1.2 (4)	C79—C80—C81—C82	1.4 (5)
C38—C39—C40—F4	179.6 (2)	C80—C81—C82—C83	1.2 (5)
C38—C39—C40—C41	0.6 (4)	C80—C81—C82—F8	−178.4 (3)
F4—C40—C41—C42	179.4 (3)	F8—C82—C83—C84	177.6 (3)
C39—C40—C41—C42	−1.6 (5)	C81—C82—C83—C84	−2.0 (5)
C40—C41—C42—C37	0.8 (4)	C82—C83—C84—C79	0.3 (5)
C38—C37—C42—C41	0.9 (4)	C80—C79—C84—C83	2.1 (5)
C36—C37—C42—C41	177.9 (3)	C78—C79—C84—C83	−179.5 (3)

### Hydrogen-bond geometry (Å, º)

**Table:**

*D*—H···*A*	*D*—H	H···*A*	*D*···*A*	*D*—H···*A*
C4—H4···O6	0.95	2.60	3.505 (4)	160
C6—H6···F7^i^	0.95	2.35	3.238 (3)	155
C18—H18···F4^i^	0.95	2.45	3.363 (4)	162
C23—H23···O2	0.95	2.56	3.504 (4)	172
C25—H25···F2^ii^	0.95	2.43	3.303 (4)	154
C28—H282···Br3^iii^	0.99	2.80	3.784 (3)	170
C31—H31···O5^iii^	0.95	2.66	3.557 (5)	157
C36—H362···Br2^iv^	0.99	2.97	3.933 (3)	164
C39—H39···O3^v^	0.95	2.44	3.373 (4)	166
C44—H44···F8^vi^	0.95	2.48	3.341 (4)	150
C46—H46···Br3^ii^	0.95	2.79	3.708 (3)	164
C52—H52···O9^iv^	0.95	2.53	3.292 (6)	138
C60—H60···F8^vii^	0.95	2.55	3.329 (4)	139
C62—H62···O9^viii^	0.95	2.48	3.365 (6)	156
C65—H65···Br4^viii^	0.95	2.91	3.653 (3)	136
C67—H67···O2^iv^	0.95	2.58	3.503 (4)	165
C69—H69···Br1^iv^	0.95	3.07	3.750 (3)	130
C73—H73···F1^ii^	0.95	2.60	3.312 (4)	132
C81—H81···F5^ix^	0.95	2.50	3.436 (4)	169
C83—H83···O1^x^	0.95	2.52	3.263 (4)	135
O1—H111···Br4^xi^	0.89 (2)	2.57 (2)	3.449 (3)	167 (3)
O1—H112···Br4	0.88 (2)	2.61 (2)	3.476 (3)	170 (3)
O2—H211···Br3	0.89 (2)	2.50 (2)	3.384 (3)	168 (3)
O2—H212···Br1	0.87 (2)	2.44 (2)	3.317 (2)	178 (3)
O3—H311···Br2	0.90 (2)	2.39 (2)	3.274 (2)	169 (3)
O3—H312···Br1	0.89 (2)	2.44 (2)	3.307 (2)	164 (3)
O4—H411···O5	0.95 (2)	2.12 (2)	3.033 (5)	161 (4)
O4—H412···Br1	0.93 (2)	2.32 (2)	3.250 (3)	178 (4)
O5—H511···O3	0.88 (2)	1.93 (2)	2.809 (4)	172 (5)
O5—H512···O7^xii^	0.90 (2)	2.11 (3)	2.906 (4)	147 (4)
O6—H611···Br2^iv^	0.86 (2)	2.57 (2)	3.407 (2)	165 (3)
O6—H612···Br2^iii^	0.87 (2)	2.44 (2)	3.306 (3)	173 (4)
O7—H711···O6	0.90 (2)	2.03 (2)	2.925 (4)	171 (4)
O8—H811···O9	0.94 (2)	2.45 (3)	3.358 (6)	163 (4)
O8—H812···Br4	0.94 (2)	2.71 (3)	3.579 (3)	154 (4)

Symmetry codes: (i) −*x*+2, −*y*, −*z*+1; (ii) −*x*+1, −*y*, −*z*+1; (iii) *x*+1, *y*, *z*; (iv) −*x*+1, −*y*+1, −*z*+1; (v) *x*+1, *y*−1, *z*; (vi) −*x*+1, −*y*, −*z*+2; (vii) −*x*+2, −*y*, −*z*+2; (viii) −*x*+2, −*y*+1, −*z*+1; (ix) −*x*+1, −*y*+1, −*z*+2; (x) *x*, *y*−1, *z*+1; (xi) −*x*+2, −*y*+1, −*z*; (xii) *x*−1, *y*, *z*.
