# Supplementary figures and images for: Crystal structure of bis(bis{(E)-[(6-{(E)-[(4-fluorobenzyl)imino]methyl}pyridin-2-yl)methylidene](4-fluorophenyl)amine}nickel(II)) tetra­bromide nona­hydrate
Source: Acta Crystallogr E Crystallogr Commun. 2015 Nov 21;71(Pt 12):m226–7. doi: 10.1107/S2056989015021519 (PMC4719846; doi:10.1107/S2056989015021519)

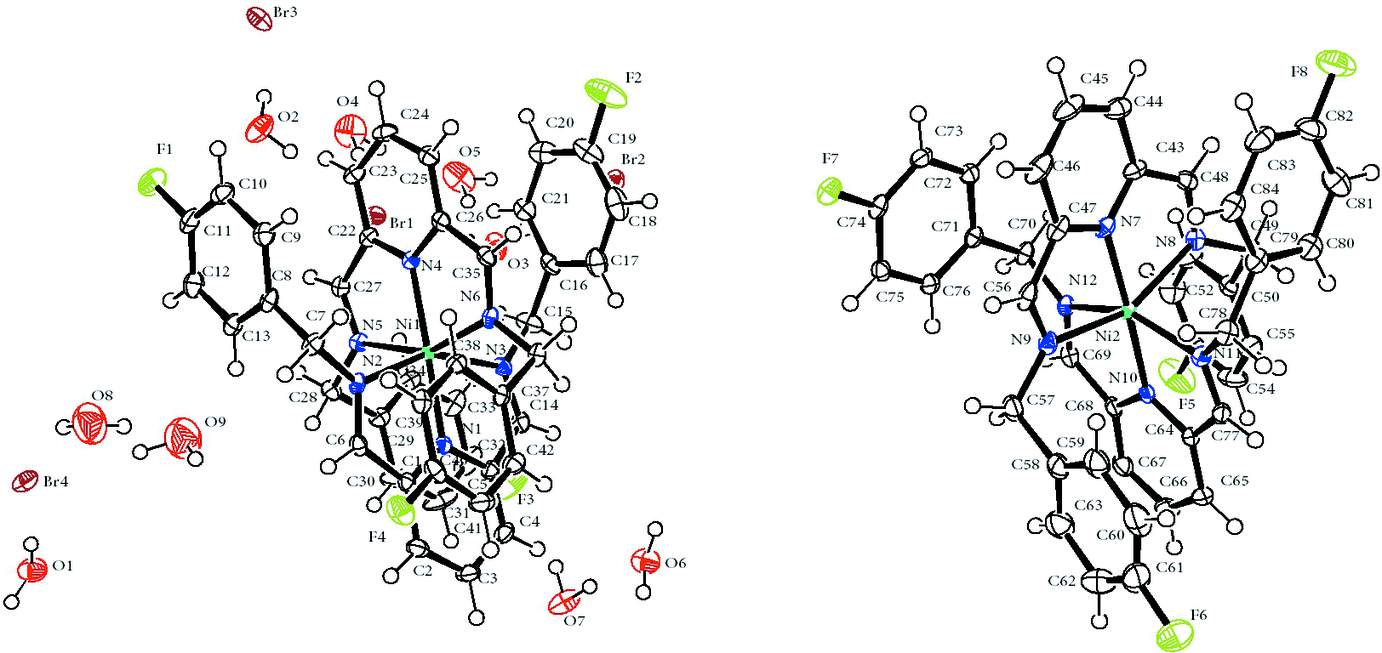

Supplement: Supplementary file 3 [file e-71-0m226-fig1.tif]

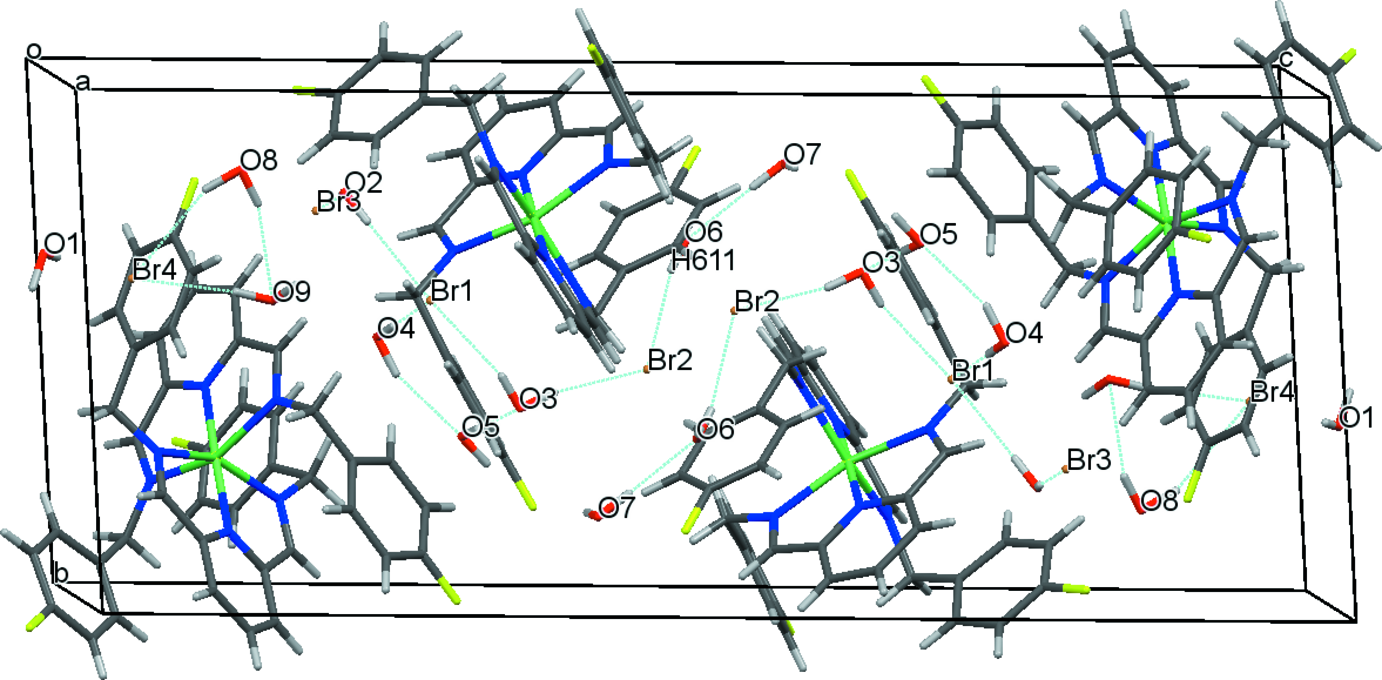

Supplement: Supplementary file 4 [file e-71-0m226-fig2.tif]

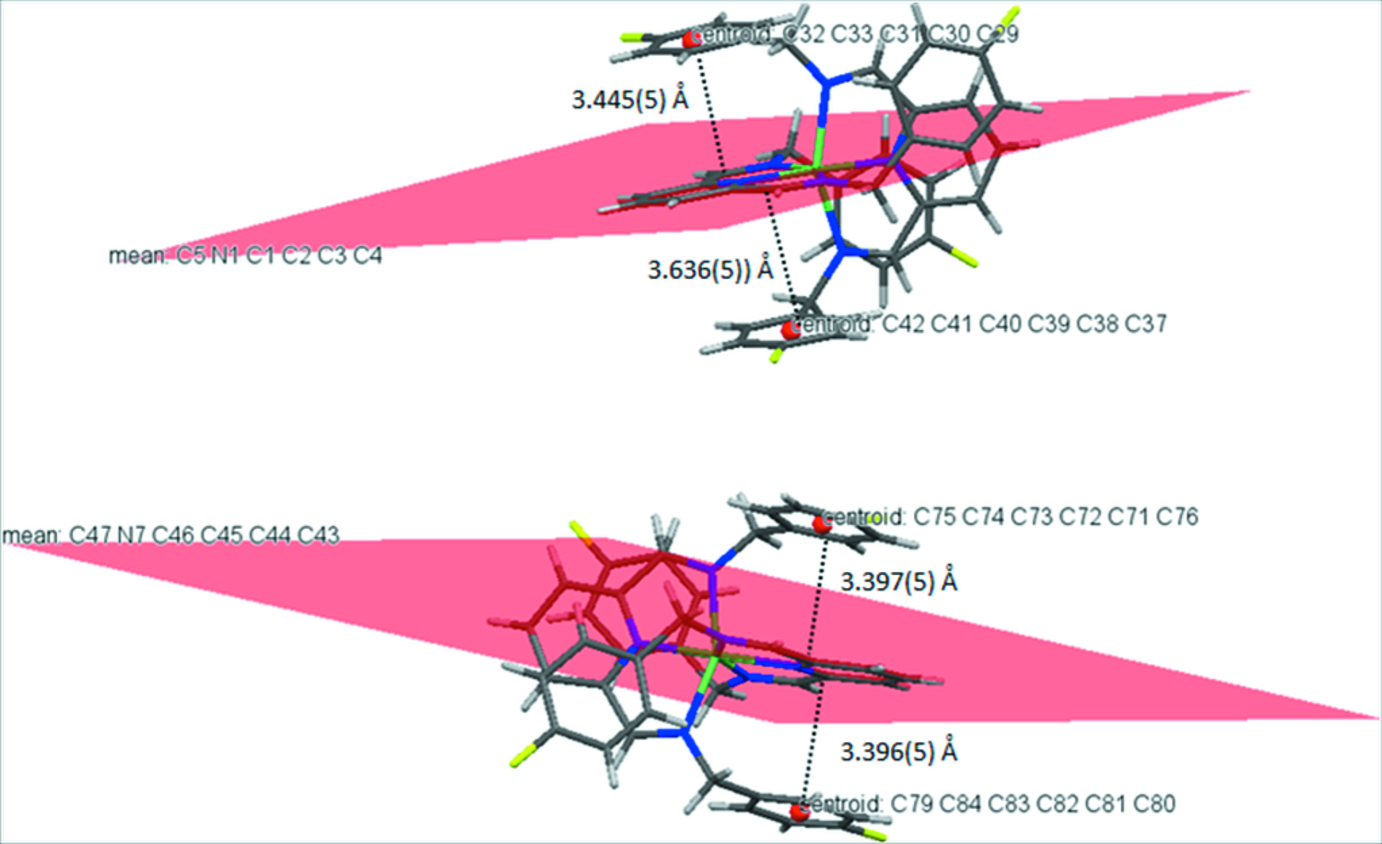

Supplement: Supplementary file 5 [file e-71-0m226-fig3.tif]
